# Molecular Dynamics in Tetrafluoridoborate Salts of Divalent Metals Studied by Nuclear Magnetic Resonance Spectroscopy

**DOI:** 10.1002/cphc.202500721

**Published:** 2026-03-05

**Authors:** Anton Gradišek, Kristian Radan, Matic Lozinšek

**Affiliations:** ^1^ Jožef Stefan Institute Ljubljana Slovenia

**Keywords:** fluorides, metal tetrafluoridoborates, molecular dynamics, nuclear magnetic resonance spectroscopy (NMR)

## Abstract

Fluoridoborate (BF_4_^−^) salts are linchpins across synthesis and catalysis, key constituents of ionic liquids, and promising electrolytes and additives in next‐generation multivalent rechargeable batteries. Despite this reach, quantitative links between crystal chemistry and anion motion remain scarce. Here, we present a molecular dynamics study of four such salts, namely M(BF_4_)_2_ (M = Ca^2+^, Sr^2+^, Ba^2+^, Cd^2+^), by means of nuclear magnetic resonance spectroscopy (NMR). We measured the temperature dependence of static ^19^F NMR spectra and spin‐lattice relaxation and determined the values of activation energies for the thermally‐activated reorientations for the BF_4_^−^ tetrahedra. We draw a comparison with systems with similar geometries, such as the well‐studied complex metal borohydrides.

## Graphical abstract

We studied molecular dynamics in four tetrafluoridoborate salts by means of nuclear magnetic resonance. Characteristical motions in these systems are thermally‐activated reorientations of BF_4_^−^ ions. We determine the activation energies for these processes and compare the dynamics with those in borohydride systems that share the tetrahedral geometry of the anions.

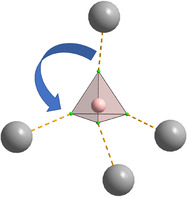

## 1 Introduction

Tetrafluoridoborate anion, BF_4_^−^, is the smallest representative of the weakly coordinating anions [1]. As a versatile and ubiquitous chemical species, it is employed in numerous salts for the preparation of cationic reagents [2], catalysts [3], ionic liquids [4], and to stabilise and isolate electrophilic cations [1]. Furthermore, the BF_4_^−^ salts are utilised in battery research. For example, a Ca(BF_4_)_2_‐based electrolyte was employed in the first demonstration of calcium plating and stripping on a metallic calcium electrode [5].

Nuclear magnetic resonance (NMR) is a powerful spectroscopic technique, well suited to study different types of dynamic processes in solids. Apart from direct measurements of self‐diffusion using pulse gradient spin echo (PGSE) or related methods, temperature dependencies of NMR spectra and relaxation times can provide insight into local dynamics, such as reorientations of smaller units.

Surprisingly, there have not been many studies on the molecular dynamics of BF_4_^−^ ions using solid‐state NMR methods. Anion motion in the tetrafluoridoborate salts of monovalent cations MBF_4_ (M = NH_4_^+^, ND_4_^+^, K^+^, Rb^+^, Cs^+^, NO^+^, NO_2_^+^) was studied previously by ^19^F NMR relaxation and linewidths measurements, which evidenced BF_4_^−^ tumbling motion [6, 7]. Moreover, the orientational disorder of the cation in the nitrosonium tetrafluoridoborate, NOBF_4_, was observed in the crystal structure [8] and studied by heat capacity measurements in the temperature range from 10 to 304 K [9].

Motions of [Sb(CH_3_)_4_]X, X = PF_6_^−^, BF_4_^−^ were studied by means of ^19^F second moments to identify tumbling motions of the cations and anions [10]. Local dynamics and ionic conductivity in organic solid‐state ionic conductors containing BF_4_^−^ anions and proton‐containing cations were investigated using proton and fluorine NMR spectra [11]. Solid‐state MAS NMR spectroscopy combined with various multi‐nuclei experiments was employed to examine the immobilisation of a BF_4_^−^ salt of a palladium(II) complex on silica [12]. Proton and fluorine spectra and spin‐lattice relaxation measurements were used to study dynamics in [Mg(H_2_O)_6_](BF_4_)_2_ [13].

On the other hand, there are numerous studies using both NMR spectra and spin‐lattice relaxation times to study molecular dynamics in systems with BH_4_^−^ anions, which share the tetrahedral geometry but are much lighter than BF_4_^−^ ions. Complex metal borohydrides M(BH_4_)_*n*_ are key solid‐state hydrogen‐storage materials [14], where BH_4_^−^ reorientations govern transport and (de)hydrogenation, and a better understanding of molecular dynamics allows for the synthesis of novel materials and tuning of physio‐chemical properties. BH_4_^−^ exhibits different types of reorientations around various crystallographic axes [15, 16, 17, 18, 19], with distinct thermally‐activated energies required for each type of reorientation. Contrasting BF_4_^−^ with the lighter and isosteric BH_4_^−^ isolates how anion mass and polarizability set reorientational barriers and lattice coupling; a proton‐free, robust M(BF_4_)_2_ framework would also enable clean, nucleus‐specific NMR comparisons (^19^F vs. ^1^H/^11^B). With alkali‐metal BF_4_^−^ benchmarks in place [7], extending the comparison to alkaline‐earth (divalent) systems would test how cation valence and radius reshape BF_4_^−^ reorientational dynamics within a common anion scaffold.

This study focuses on the tetrafluoridoborate salts of divalent cations M(BF_4_)_2_ (M = Ca^2+^, Sr^2+^, Ba^2+^, Cd^2+^). We selected the first three to represent a systematic series of alkaline‐earth cations of increasing radius, while Cd(BF_4_)_2_ was included because it is isostructural with the Ca and Sr salts, enabling a direct comparison within a common lattice, while at the same time providing a d‐block analogue whose distinct electronic character allows us to probe the influence of cation polarizability on the BF_4_^−^ reorientational dynamics.

By means of temperature‐dependent ^19^F NMR spectra and spin‐lattice relaxation, we studied reorientational motions of the BF_4_^−^ units. We analyse the obtained values of the activation energies in view of the different structures of these salts and we draw a comparison with the well‐studied BH_4_‐containing systems.

## 2 Experimental Details

### 2.1 Synthesis and Characterisation

Tetrafluoridoborate salts were synthesised from the corresponding metal fluorides (CaF_2_, SrF_2_, BaF_2_, CdF_2_) and gaseous BF_3_ in anhydrous HF [20, 21, 22, 23, 24].

The syntheses were carried out under strictly anhydrous conditions. Volatile reagents were handled using a brass‐encased fluoroplastic (PTFE ‐ polytetrafluoroethylene and FEP ‐ fluorinated ethylene propylene) vacuum‐gas manifold connected to a two‐stage rotary vane pump through metal (nickel, copper) vacuum lines equipped with a soda lime scrubber and liquid nitrogen‐cooled traps. Non‐volatile materials, sensitive to traces of moisture, were stored and handled in a glovebox (Mbraun, Garching, Germany) with a maximum water content of 0.5 ppm. Custom‐made FEP reaction vessels equipped with PTFE valves and PTFE‐covered stir bars were used for the syntheses. All reaction vessels and connexions were rigorously dried under dynamic vacuum and passivated overnight with ∼1 bar of F_2_ gas.

The reagents BaF_2_ (Alfa Aesar, 99.99%), CaF_2_ (Merck, pro analysis), CdF_2_ (Alfa Aesar, 99.9%, metals basis), SrF_2_ (Alfa Aesar, 99.99%), BF_3_ (Union Carbide, 99.5%), and F_2_ (Solvay Fluor, 98–99 vol%) were used as purchased. To remove traces of water, the solvent anhydrous HF (aHF; Fluka, purum and Linde, Fluorwasserstoff 99.95%) was additionally treated with K_2_NiF_6_ (Ozark‐Mahoning, 99%) for several days prior to use. *Caution: aHF*, *BF_3_, and F_2_ must be handled in a well‐ventilated fume hood solely by experimentalists who are familiar with these hazardous chemicals and the risks associated with them. The use of protective gear is obligatory.*

The compounds M(BF_4_)_2_ (M = Ca, Sr, Ba, Cd) were synthesised in a FEP reaction vessel by a stepwise condensation of a 10%–54% stoichiometric excess of BF_3_ onto a frozen solution of the corresponding binary fluoride MF_2_ (1.49–4.19 mmol) in ca. 3.5 mL of aHF cooled to −196°C with liquid nitrogen. When the reaction mixture was warmed up to room temperature, a voluminous white precipitate formed under a clear colourless solution. The reaction vessel was then placed on a laboratory shaker and continuously agitated for 2–5 days. The excess of BF_3_ and aHF were pumped off and dried under dynamic vacuum until a constant mass of the white solid was achieved. The purity of the products was assessed by Raman spectroscopy.

The structures of the four systems were previously determined by single‐crystal X‐ray diffraction (SCXRD). Three of the compounds (M = Ca, Sr, Cd) are isostructural and crystallize in the orthorhombic space group *Pbca* (*V *= 1055.7(9)–1235.0(10) Å^3^; *Z *= 8) [24, 25, 26], whereas Ba(BF_4_)_2_ adopts monoclinic *C*2/*m* space group with a smaller unit cell (*V *= 277.3(2) Å^3^; *Z *= 2) [23].

**FIGURE 1:**
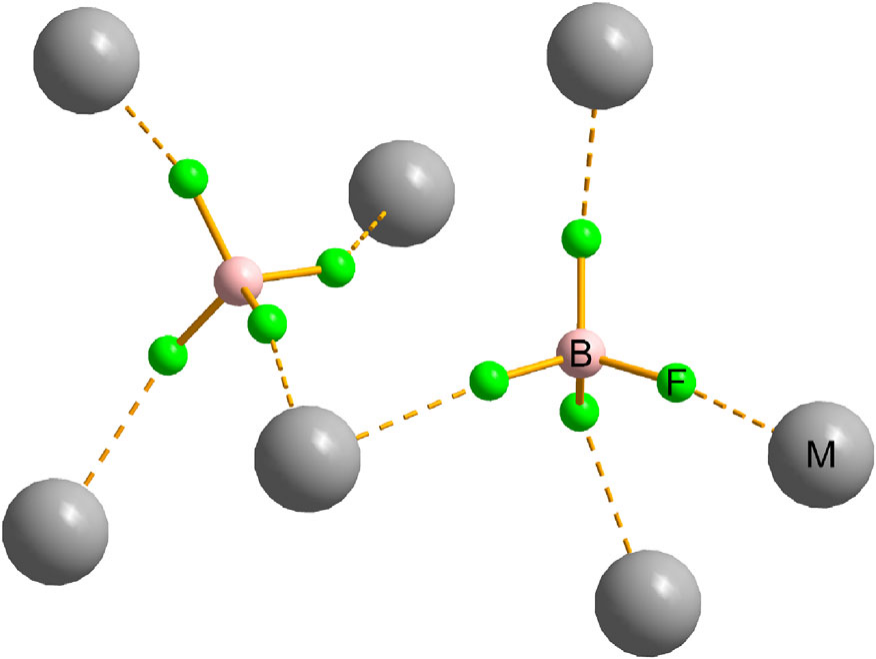
BF_4_^−^ coordination spheres in the structures of M(BF_4_)_2_ (M = Ca, Sr, Cd). Two crystallographically independent BF_4_^−^ anions engage in eight M–F contacts to the nearest M^2+^ centres with contact distances (Å): 2.330(2), 2.345(1), 2.347(1), 2.354(2), 2.360(2), 2.367(2), 2.396(2), 2.401(2) (M = Ca) [25]; 2.490(4), 2.495(4), 2.496(4), 2.502(4), 2.506(4), 2.507(4), 2.529(4), 2.538(4) (M = Sr) [26]; 2.296(2), 2.300(3), 2.306(3), 2.308(3), 2.334(2), 2.336(3), 2.351(2), 2.381(3) (M = Cd) [24].

**FIGURE 2:**
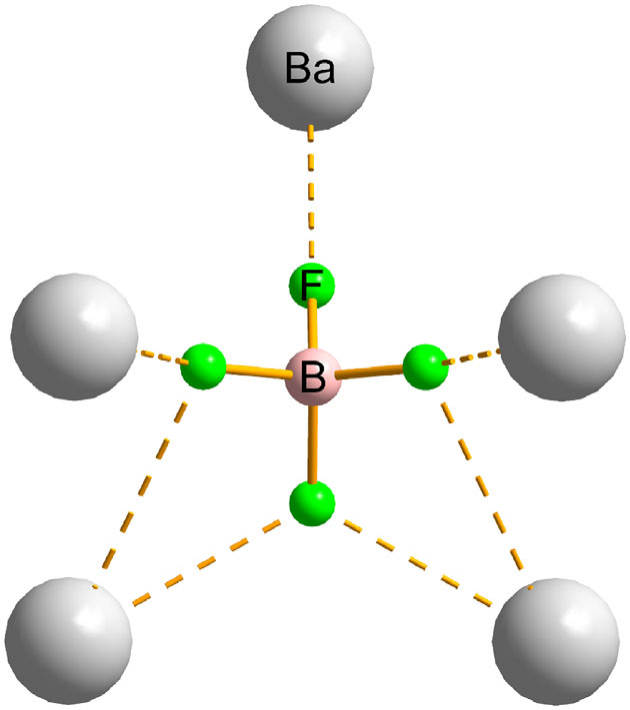
BF_4_^−^ coordination environment in the structure of Ba(BF_4_)_2_. The depicted Ba–F contacts to the closest Ba^2+^ centres comprise seven interactions (Å): 2.690(5), 2 × 2.765(3), 2 ×  2.886(2), and two longer contacts at 3.361(5) [23].

The coordination environments of the crystallographically independent BF_4_^−^ anions are shown in Figures 1 and 2. In Ba(BF_4_)_2_, each BF_4_^−^ unit coordinates to five nearest Ba^2+^ ions, whereas in the other three systems it coordinates to four nearest metal ions. The F–M distances determined by SCXRD range from ≈2.3 to 3.4 Å; only the contacts within the primary coordination sphere are considered. The average F–M distances follow the trend Ca ≈ Cd < Sr < Ba.

### 2.2 Nuclear Magnetic Resonance Measurements

Samples were sealed in glass tubes under an Ar atmosphere to prevent contact with water vapour or oxygen. They were stored in dark to prevent light exposure. ^19^F NMR spectra and spin‐lattice relaxation times were measured as a function of temperature in a heating run. The measurements were conducted at a superconducting magnet with the field of 2.35 T, corresponding to Larmor frequency *ν*_L_(^19^F) = 94.08 MHz with a custom in‐house made probe and an in‐house made spectrometer. Temperature was controlled using a gas‐flow cryostat with an Oxford ITC4 Intelligent Temperature Controller. Static NMR spectra were recorded using 90_*x*_ − *τ* − 90_*y*_ spin echoes, and spin‐lattice relaxation times were measured using the saturation‐recovery pulse sequence. A 90° pulse of 4.5 μs was used. Data was recorded using a 0.25 μs dwell time in 1024 points. Individual parameters for the spectra and *T*_1_ measurements were optimised according to the particular experiment, as the *T*_1_ values varied widely with the changing temperature.

## 3 Results and Discussion

### 3.1 NMR Spectra

**FIGURE 3:**
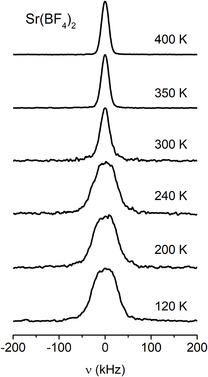
Static ^19^F NMR spectra of Sr(BF_4_)_2_, measured at 2.35 T (94.08 MHz), at some selected temperatures.

**FIGURE 4:**
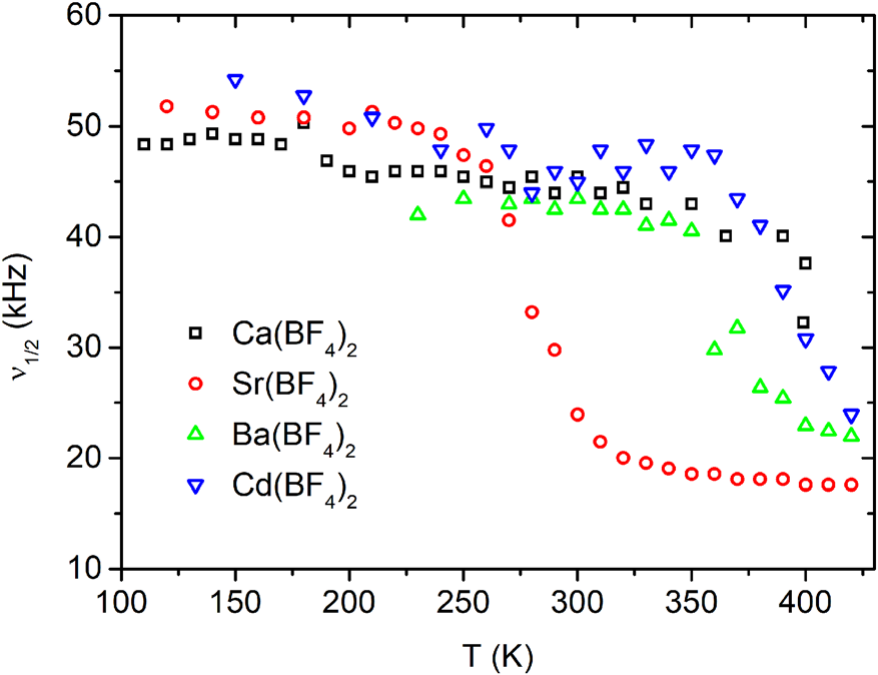
Temperature dependencies of the width of the static ^19^F spectra (full width at half‐maximum), measured at 2.35 T, for all four systems.

Figure 3 shows the evolution of the ^19^F spectra in Sr(BF_4_)_2_, which can be viewed as the representative example, upon heating. At the lowest temperatures in the experiment, the NMR line is broad and has a roughly Gaussian line shape. Upon heating, the line gets narrower, although the shape still remains Gaussian. The line shape transition takes place roughly between 250 and 300 K, as seen in Figure 4 where we show the temperature dependence of line width ($\nu_{1/2}$, full width at half maximum). The behaviour trend is the same for all four samples, the difference being the temperature range when the line narrowing occurs (above 350 K for the other three systems).

The fluorine line shape is predominantly determined by the nuclear magnetic dipole–dipole interactions, both homonuclear (fluorine–fluorine) and heteronuclear (fluorine–boron or one of the cations). A small contribution to the line width originates from the inhomogeneity of the magnetic field. In the investigated systems, we can look at the ‘intramolecular' and ‘intermolecular' dipolar interactions. The former are the interactions within an individual BF_4_^−^ unit where the interatomic distances remain the same, but the angle between the vector connecting two nuclei and the direction of the external magnetic field can change in case of motions. The latter, the intermolecular interactions, take place between separate groups. The onset of molecular motions causes the averaging of both types of interactions. In case of isotropic reorientational motions, like in a liquid, the intramolecular interactions are averaged to zero. In case of anisotropic motions, such as rotations about a fixed axis, the interactions are only partially averaged. On the other hand, such reorientational motions will only partially average the intermolecular interaction.

In all four investigated systems, the narrowing of the fluorine spectra can be associated with the onset of reorientations of BF_4_^−^ tetrahedra. At this point, we can draw a comparison with borohydride systems, such as α‐$\mathrm{Mg}{\left({\mathrm{BH}}_{4}\right)}_{2}$ [15] or LiZn_2_(BH_4_)_5_ [18]. The location of the BH_4_ tetrahedra symmetrically between two metal atoms allows for three types of reorientational motions: (i) rotations around the twofold axis (*C*_2_) along the line that connects the two metal atoms (M)—here, the distances between H and M remain unchanged, thus this process has the lowest activation energy, (ii) rotations around the threefold axis (*C*_3_) where one of the H—M bonds remains fixed and other three break, and (iii) rotations around the twofold axis (*C*_2_) perpendicular to the M–M line, where all the M–H bonds are broken (this process requires the highest activation energy). In less symmetric systems, such as in Sr(BH_4_)_2_(NH_3_)_2_ [19], where the BH_4_ tetrahedron is located at the apex of an isosceles triangle with two Sr atoms, there are two types of rotations, corresponding to modes (ii) and (iii) from above. In systems investigated in this study, the BF_4_^−^ units coordinate to four or five nearest atoms instead of two, thus the anion is stronger bound and the reorientational axes are less clearly defined.

At the highest temperatures in our study, the fluorine spectra in all four systems still retain a Gaussian shape, indicating that the interactions did not average completely (which would result in a Lorentzian shape). Thus, the studied compounds remained in the regime where none of the BF_4_^−^ groups detach from the structure and move around freely.

Comparing the results to the related work, the line narrowing for all our samples takes place at much higher temperatures than in the case of monovalent cations [7].

### 3.2 Spin–Lattice Relaxation

**FIGURE 5:**
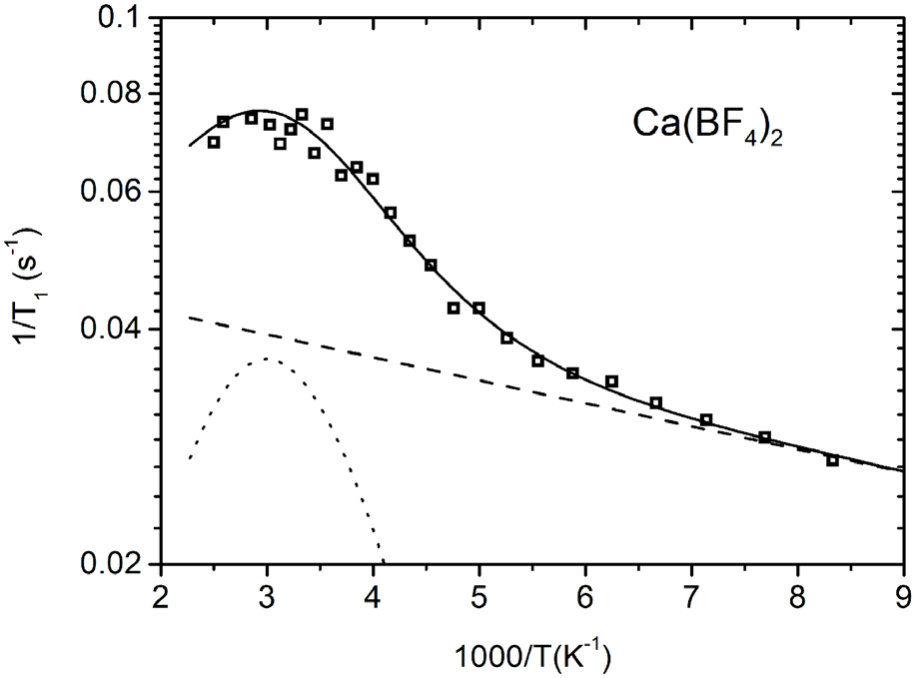
Temperature dependence of spin–lattice relaxation rate for ^19^F in Ca(BF_4_)_2_. Solid line represents the model fit and is the sum of both contributions: dash line is the background and the dotted line is the BPP‐like model related to the reorientations of BF_4_^−^ units.

**FIGURE 6:**
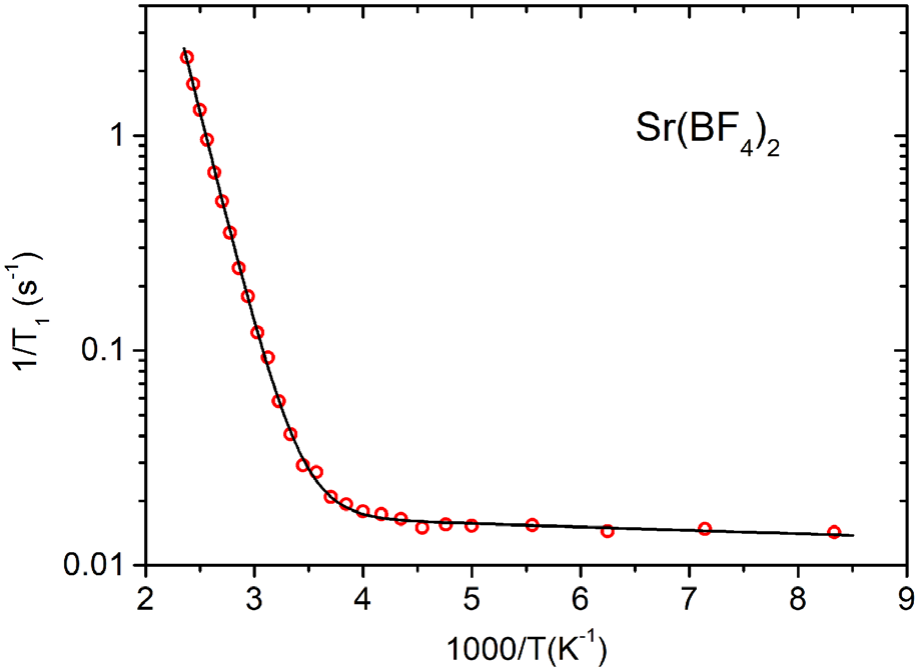
Temperature dependence of spin–lattice relaxation rate for ^19^F in Sr(BF_4_)_2_. Solid line represents the model fit.

**FIGURE 7:**
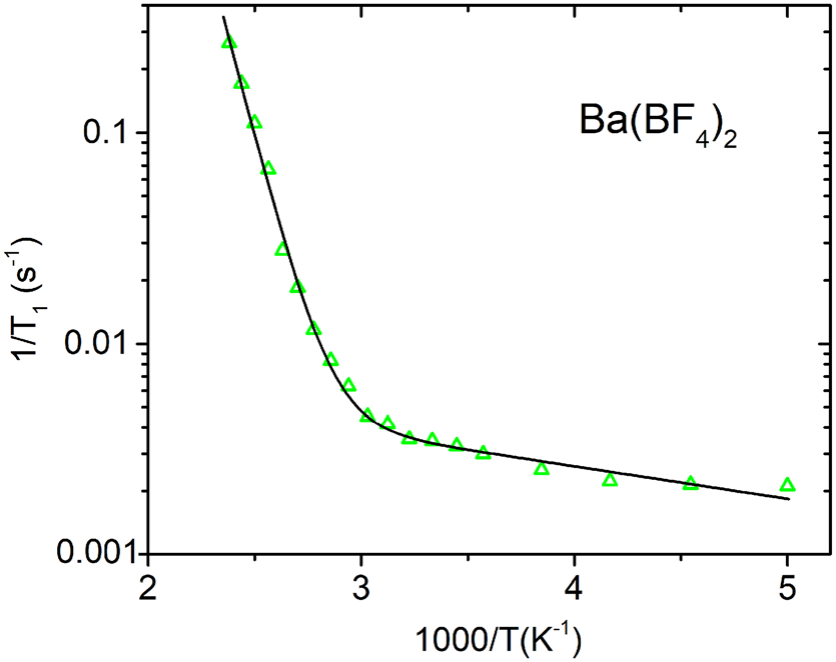
Temperature dependence of spin–lattice relaxation rate for ^19^F in Ba(BF_4_)_2_. Solid line represents the model fit.

**FIGURE 8:**
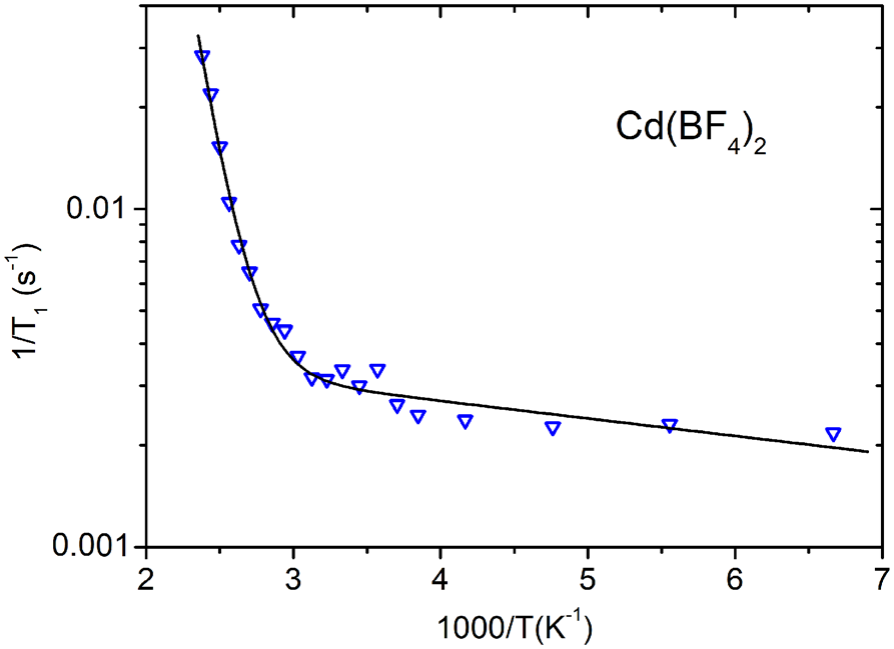
Temperature dependence of spin–lattice relaxation rate for ^19^F in Cd(BF_4_)_2_. Solid line represents the model fit.

Figures 5–8 show the ^19^F spin–lattice relaxation rates (defined as $R_{1}=1/T_{1}$), measured at 2.35 T, as a function of inverse temperature. Sr(BF_4_)_2_, Ba(BF_4_)_2_, and Cd(BF_4_)_2_ all show the same trend, with *R*_1_ changing little at low temperatures while starting to increase rapidly above a certain temperature. In Ca(BF_4_)_2_, the *R*_1_ levels off at the highest temperatures and then shows signs of decreasing, with a peak at around 330 K. The relaxation data were reasonably fitted using a single exponential function, meaning that all fluorine atoms in the sample relax with the same rate.

While the fluorine NMR spectra are determined by the dipolar spin interactions, the spin–lattice relaxation is governed by the fluctuations of these interactions, both homonuclear and heteronuclear. For the homonuclear part (F–F), we use the standard relaxation model, developed by Bloembergen, Purcell, and Pound (BPP) [27]. This model assumes an exponential correlation function for random dipolar field fluctuation, which is characterised by a single correlation time. This part of relaxation is expressed as

(1) $$\begin{array}{c} R_{1}^{F}=\frac{2\Delta M_{\mathrm{FF}}}{3}\left[\frac{\tau }{1+\omega_{F}^{2}\tau^{2}}+\frac{4\tau }{1+4\omega_{F}^{2}\tau^{2}}\right] \end{array}$$

where Δ*M*_FF_ is the fluctuating part of the second moment due to F–F dipolar interaction. *ω*_F_ = 2*πν*_F_ is the fluorine Larmor frequency. *τ* ^−1^ is the fluorine jump rate (inverse of correlation time) for the rotation and is assumed to have an Arrhenius‐line temperature dependence

(2) $$\begin{array}{c} \tau =\tau_{0}\exp\;\left(E_{a}/k_{B}T\right) \end{array}$$

where $\tau_{0}^{-1}$ is the attempt frequency and *E*_a_ is the activation energy for the process.

For the heteronuclear contribution to relaxation, we follow the formalism of Abragam for spins I and S [28]:

(3) $$\begin{array}{cc} R_{1}^{IS} & =\frac{\Delta M_{\mathrm{IS}}}{2}[\frac{\tau }{1+{\left(\omega_{I}-\omega_{S}\right)}^{2}\tau^{2}}+\frac{3\tau }{1+\omega_{I}^{2}\tau^{2}}\\ & +\frac{6\tau }{1+{\left(\omega_{I}+\omega_{S}\right)}^{2}\tau^{2}]} \end{array}$$

Here, fluorine takes the place of spin I and other atoms are S, then all contributions are summed (one should mention that the F–B dipolar coupling is considerably stronger than that of F with other metallic atoms in our systems). Δ*M*_IS_ is the fluctuating part of the second moment due to I–S interaction, while the *ω*_*i*_ are the corresponding Larmor frequencies. Total relaxation is the sum of the homonuclear and heteronuclear contributions. In case there is more than one rotation mode (as discussed above, there can even be three modes with different activation energies [18]), each contribution has to be added separately with corresponding *E*_a*i*_ and *τ*_0*i*_.

The above models produce a maximum in *R*_1_ in the region where *ωτ *≈ 1. Far from this maximum, in the so‐called fast‐motion approximation (*ωτ *≪ 1) or in the slow‐motion approximation (*ωτ *≫ 1), the relaxation can be simplified to the asymptotic form as

(4) $$\begin{array}{c} R_{1}=A\exp\;\left(\pm \frac{E_{a}}{k_{B}T}\right) \end{array}$$

where $A\approx 1/\omega^{2}\tau_{0}$ for *ωτ *≫ 1 and *A *≈ *τ*_0_ for *ωτ *≪ 1.

In addition, another contribution becomes prominent at low temperatures. Often, in that temperature range, relaxation levels off due to interactions with paramagnetic impurities that are unavoidably present in the sample in small concentrations [18, 29]. This contribution is usually treated as temperature‐independent, however, here it appears to have a slight slope. A possible explanation is tumbling motions of a small fraction of units, either in locally distorted environments or due to impurities [29]. To describe this additional process, we use Equation (4) with a small activation energy.

We now use the above models to analyse the spin–lattice relaxation data for all four samples. Fitting the relaxation models to the experimental data was performed using a non‐linear least‐squares minimisation with a global minimum target [30]. Sr(BF_4_)_2_, Ba(BF_4_)_2_, and Cd(BF_4_)_2_ do not exhibit a maximum in relaxation, thus we use the model for the slow‐motion relaxation limit, Equation (4), with the prefactor *A*_1_ and the activation energy for rotations/reorientations of the BF_4_^−^ tetrahedra *E*_a1_. Evidently, as there are no peaks in relaxation, we are in the slow motional regime (*ωτ *≪ 1), and since there is a single slope value in the high‐temperature range, we are looking at only one reorientational mode with a single activation energy. In Ca(BF_4_)_2_, a weak peak appears at higher temperatures. Here, we attempt to estimate the activation energy and the correlation time, in order to keep the number of fitting parameters as small as possible. Therefore, we use a BPP‐like model (Equation (1)) with a prefactor *M* and parameters *E*_a_ and *τ*_0_. Looking at the relaxation data at low temperatures, we see that the relaxation rate is increasing slightly in all four systems. We describe this part using the limit model (Equation (4)) with a low activation energy *E*_a2_ and a prefactor *A*_2_.

Model fit parameters for all four systems are listed in Table 1 and model curves are shown on Figures 5, 6, 7, 8. In a big picture, the relaxation behaviour of the systems is consistent with what was observed for tetrafluoridoborate salts of monovalent cations [6, 7]. However, notably, in our series of alkaline‐earth tetrafluoridoborates, the activation energy for BF_4_^−^ reorientation increases with cation size. This trend is opposite to that observed for alkali‐metal analogues [7], where larger monovalent cations lower the reorientational barrier. We attribute the reversal to stronger, more directional electrostatic binding with divalent cations (multidentate for Ba^2+^), together with packing and coordination motifs that curtail the free volume available for anion motion. In monovalent cations, the activation energies obtained for reorientations range from ≈500 meV for Na^+^ to ≈300 meV for Cs^+^. While the values for divalent cations are of the same order of magnitude, Ca^2+^ sticks out with a much lower activation energy than other systems. The reason for this is unclear but could be attributed to a higher disorder in the sample, resulting in lowering of the reorientational barriers.

**TABLE 1:** Model fit parameters for all four systems, specific models are discussed in the text. *E*_a*i*_ are the activation energies for the two dynamic processes (the value in italic is in kJ/mol), *A*_i_ are the prefactors for the limit case (Equation (4)), while *M* and *τ*_0_ are the parameters of the BPP‐like model (Equation 1).

	*E*_a1_, meV, kJ/mol	*A*_1_, s^−1^	*E*_a2_, meV, kJ/mol	*A*_2_, s^−1^	*M*, s^−2^	*τ*_0_, s^−1^
Ca	100(10), *10*	—	6(1), *0.6*	4.8(1) × 10^−2^	1.5(1) × 10^7^	3(1) × 10^−11^
Sr	405(4), *39*	1.6(1) × 10^5^	3.1(1), *0.3*	1.9(1) × 10^−2^	—	—
Ba	770(10), *75*	5(1) × 10^8^	31(1), *3*	1.1(1) × 10^−2^	—	—
Cd	535(20), *52*	6(2) × 10^4^	10(1), *1*	4.3(1) × 10^−3^	—	—

Returning again to the comparison between the tetrafluoridoborates and borohydrides, we see that we cannot identify different reorientational modes of the BF_4_^−^ units using our relaxation model, as opposed to the system with much lighter borohydride tetrahedra [15, 18, 19]. Partially, as discussed in the analysis of the NMR spectra, this is due to different geometries, with the BF_4_^−^ units being coordinated to four or five nearest metal atoms. Moreover, in the tetrafluoridoborate systems, the peak in relaxation rate is observed only in the Ca(BF_4_)_2_, while the relaxation is still in the slow‐motion regime in the other three systems. Measurements at higher temperatures, required to reach a peak in relaxation rates, are unfeasible as they might result in a thermal decomposition of the samples. Thus, only a single thermally‐activated reorientational mode can be identified for each of the systems.

We can draw an additional parallel with the NMR studies of decaboranes and dodecaboranes, such as Ag_2_B_12_H_12_ [29] and (NH_4_)_2_B_10_H_10_ and (NH_4_)_2_B_12_H_12_ [31, 32], where large boron cages can, in principle, move around different crystallographic axes; however, only a single reorientational mode was identified from the NMR relaxation measurements. Similarly to what we observed in the relaxation curves at lower temperatures for all tetrafluoridoborate samples, the analysis of the borohydride tetrahedra or cages also exhibited additional dynamic processes with low activation energies, which were linked to tumbling motions, impurities, or local disorder [18, 19, 29].

## 4 Conclusion

We studied molecular dynamics in four tetrafluoridoborate salts by means of NMR spectroscopy, in three salts with alkaline‐earth cations and in Cd(BF_4_)_2_, which is isostructural with Ca and Sr salts. The analysis of the temperature dependence of the ^19^F NMR spectra demonstrated the onset of reorientations of the BF_4_^−^ anions, which cause the narrowing of the spectra upon heating. These motions are thermally activated and the associated activation energies were obtained through the analysis of the temperature dependence of the ^19^F spin–lattice relaxation rates. The values are of the same order of magnitude as those obtained previously for a series of monovalent cations, although there is no clear trend in view of the cation size. Especially curious is the case of Ca with an unexpectedly low activation energy of 100 meV. In all four systems, an additional dynamic process was identified in the low‐temperature range, likely associated with tumbling motions or minute concentrations of impurities in the samples. In view of comparison with borohydride‐based materials, where different reorientational modes were previously observed, only a single mode was identified in each of the systems investigated in this study. This is attributed to a much larger size of the BF_4_^−^ anion and to different coordinations to the surrounding metal cations. Together, the results indicate that both the cation valence and the coordination multiplicity influence the mobility of the BF_4_^−^ ions. Our findings provide insight into the rational design of new materials and application‐specific optimisation for next‐generation energy‐storage and catalytic systems.

## Funding

This work was partially supported by the Slovenian Research and Innovation Agency (ARIS), basic core funding (P2‐0209), the Marie Curie International Outgoing Fellowship (628726) within the 7th European Community Framework Programme, and the European Research Council (ERC) under the European Union's Horizon 2020 Research and Innovation Programme (Starting Grant 950625).

## Conflicts of Interest

The authors declare no conflicts of interest.

## Data Availability

The data that support the findings of this study are openly available in the Zenodo repository at https://doi.org/10.5281/zenodo.18242628.
